# Ischemic Stroke and Autophagy: The Roles of Long Non-Coding RNAs

**DOI:** 10.2174/1570159X22666240704123701

**Published:** 2024-07-04

**Authors:** Longqiang Ouyang, Wenyan Xia, Ameen Abdulhasan Al-Alwany, Reena Gupta, Ibrokhim Sapaev, Sami G. Almalki, Saud Almawash, Rand Ali Ziyad, Ahmed Hussien Alawadi, Ali Alsalamy

**Affiliations:** 1 Department of Neurosurgery, The First Affiliated Hospital, Gannan Medical University, Ganzhou, Jiangxi, China;; 2 Department of Endocrinology, The First Affiliated Hospital of Gannan Medical University, Ganzhou, Jiangxi, China;; 3 Department of Medicine, College of Medicine, Baghdad University, Baghdad, Iraq;; 4 Institute of Pharmaceutical Research, GLA University, Mathura, Uttar Pradesh, 281406, India;; 5 New Uzbekistan University, Tashkent, Uzbekistan;; 6 School of Engineering, Central Asian University, Tashkent 111221, Uzbekistan;; 7 Tashkent Institute of Irrigation and Agricultural Mechanization Engineers, 39, Kari Niyaziy Str., 100000, Uzbekistan;; 8 Department of Medical Laboratory Sciences, College of Applied Medical Sciences, Majmaah University, Majmaah 11952, Saudi Arabia;; 9 Department of Pharmaceutical Sciences, College of Pharmacy, Shaqra University, Shaqra, Saudi Arabia;; 10 College of Pharmacy, National University of Science and Technology, Dhi Qar, Iraq;; 11 College of Technical Engineering, The Islamic University, Najaf, Iraq;; 12 College of Technical Engineering, The Islamic University of Al Diwaniyah, Iraq;; 13 College of Technical Engineering, The Islamic University of Babylon, Iraq;; 14 College of Technical Engineering, Imam Ja’afar Al‐Sadiq University, Al‐Muthanna 66002, Iraq

**Keywords:** Ischemic stroke, autophagy, long non-coding RNAs, pathogenesis, treatment, potential therapeutic targets

## Abstract

Ischemic stroke is a significant cause of morbidity and mortality worldwide. Autophagy, a process of intracellular degradation, has been shown to play a crucial role in the pathogenesis of ischemic stroke. Long non-coding RNAs (lncRNAs) have emerged as essential regulators of autophagy in various diseases, including ischemic stroke. Recent studies have identified several lncRNAs that modulate autophagy in ischemic stroke, including MALAT1, MIAT, SNHG12, H19, AC136007. 2, C2dat2, MEG3, KCNQ1OT1, SNHG3, and RMRP. These lncRNAs regulate autophagy by interacting with key proteins involved in the autophagic process, such as Beclin-1, ATG7, and LC3. Understanding the role of lncRNAs in regulating autophagy in ischemic stroke may provide new insights into the pathogenesis of this disease and identify potential therapeutic targets for its treatment.

## 1 INTRODUCTION

The incidence of cerebrovascular disease is predicted to rise significantly, with stroke currently the second leading cause of death worldwide. Globally, 1 in 6 people will experience a stroke in their lifetime, with more than 13.7 million people having a stroke each year and 5.5 million people dying from it [1-3]. The World Health Organization (WHO) defines a stroke as: “rapidly developed clinical signs of focal (or global) disturbance of cerebral function, lasting more than 24 hours or leading to death, with no apparent cause other than of vascular origin” [4]. It occurs when a blood vessel ruptures or the brain's blood supply is interrupted. Typically, atherosclerotic plaques are destroyed and thrombosis is induced, resulting in ischemic stroke [5]. Currently, thrombolytic therapy is a common clinical therapeutic strategy for acute ischemic stroke, and the only drug approved for clinical use in association with thrombolytic therapy is recombinant tissue plasminogen activator (rt-PA), which has a limited therapeutic window with bleeding risks [6]. Therefore, it is critical to discover novel and effective therapeutic targets for the treatment of ischemic stroke.

Several molecular processes, such as inflammation, mitochondrial dysfunction, calcium overload, excitotoxicity, acidosis, oxidative stress, and programmed cell death, are involved in ischemic brain injury [7]. Necrosis and apoptosis are important factors in ischemia-induced neuronal cell death, according to earlier research [8]. Moreover, recent studies found that autophagy is upregulated after ischemic stroke and might be involved in disease development [9]. Although the relationship between autophagy and cerebral ischemia is debatable (that is, whether it is beneficial or harmful), the significance of autophagy in various pathological and biological processes is unquestionable. Autophagy activation may worsen ischemic injury and result in cell death. On the other hand, the activation of autophagy promotes cell survival during an ischemic stroke by removing necrotic substances [10]. Although autophagy-associated signaling pathways are promising targets for ischemic stroke treatment, it is still unknown how exactly autophagy activation contributes to ischemic events and whether it has any therapeutic value. The class of RNAs known as noncoding RNAs (ncRNAs) is transcribed from the genome, but the majority of ncRNAs do not code for functional proteins [11]. Numerous diverse ncRNAs have been discovered in recent years, and they play significant regulatory roles in the physiological and pathological processes, as well as in autophagy. A type of ncRNA is long non-coding RNAs (lncRNAs) that are transcripts. They are longer than 200 nucleotides and have limited potential to code for proteins [12]. There is evidence that lncRNAs are dysregulated during ischemic stroke [13]. In this paper, the mechanism of autophagy as well as the function and types of lncRNAs was reviewed. The study focused on how autophagy is regulated by lncRNAs and revealed the crosstalk between lncRNAs-autophagy during ischemic stroke.

## 2 AUTOPHAGY

In the intracellular catabolic degradation process, known as autophagy, cytoplasmic macromolecules, aggregated proteins, damaged organelles, as well as pathogens are transferred to lysosomes where lysosomal hydrolases break them down into ATP, sugars, fatty acids, amino acids, and nucleotides before being recycled back into the cytosol [14]. In several diseases and pathological processes, such as infection, heart diseases, cell death, aging, autoimmune diseases, cancers, and neurodegeneration, autophagy plays great pathophysiological roles. Various extracellular and intracellular conditions trigger the autophagy process in cells, including oxidative stress, nutrient starvation, and inhibitors of the mammalian target of rapamycin (mTOR), such as CCI-779 and rapamycin [15, 16]. Under growth conditions, mTOR inactivates the mammalian uncoordinated-51-like protein kinase (ULK1) complex, containing autophagy-related 13 (ATG13), ATG101, ULK1, and focal adhesion kinase (FAK) family interacting protein of 200 kD (FIP200), by mediating the phosphorylation of ATG13 and ULK1 [17]. Under energy exhaustion or starvation, mTOR disassociates from the ULK1 complex and enables it to phosphorylate FIP200 and ATG13, leading to the recruitment of the PI3K complex, containing ATG14, Beclin-1, activating molecule in BECN1-regulated autophagy protein 1 (AMBRA1), vacuolar protein sorting 34 (VPS34), and p11, to endoplasmic reticulum (ER) and subsequently the formation of phagophore and autophagy initiation. Following the phosphorylation of Beclin-1 in an ULK1-dependent manner, VPS34 is activated, which phosphorylates phosphoinositide to produce phosphatidylinositol 3-phosphate (PI3P). To form an autophagosome, PI3P recruits additional proteins, such as the double FYVE-containing protein 1 (DFCP1) and the WD-repeat protein interacting with phosphoinositide (WIPI) [18]. Furthermore, two ubiquitin-like conjugation systems are required for the maturation of the autophagosome: (1) the ATG12-ATG5 binding system that recruits ATG16L to construct a complex that mediates autophagosome elongation and (2) the microtubule-associated protein light chain 3 (LC3) conjugation system [19]. The conversion of the unconjugated form of LC3 (LC3-I) to its conjugated form of autophagosomal membrane (LC3-II) is considered a sign of autophagosome formation. Finally, the fusion of the autophagosome with the lysosome results in the degradation of the ingested cytoplasmic components [18, 20]. Fig. (**1**) represents the autophagy process and mechanism in detail.

## 3 LONG NON-CODING RNAs

Since only 2% of the human genome is translated into proteins, non-coding DNA was initially considered “junk DNA”, however after the Encyclopedia of DNA Elements (ENCODE) Project Consortium, which determined that over 80% of the human genome possesses biochemical functions, the term “junk DNA” became obsolete [21]. Non-coding RNAs are classified as microRNAs (miRNA), small nuclear RNA (snRNA), PIWI-interacting RNA (piRNA), endogenous small-interfering RNA (endo-siRNA), and lncRNAs [11]. Numerous lncRNAs are produced by the extensive transcription of genomes [22]. This broad definition includes a sizable and extremely diverse collection of transcripts, each with genomic origin and a unique biogenesis. Human GENCODE statistics indicate that there are more than 16,000 lncRNA genes within the human genome, but others propose the number at over 100,000 [23]. Similar to mRNAs, lncRNAs are generally transcribed by RNA polymerase II (pol II) and structurally have 7-methyl guanosine (m7G) cap at their 5′ ends and polyadenylated (polyA) at their 3′ ends and are spliced [22]. On the other hand, lncRNAs are often more cell-specific than mRNAs and exhibit more restricted expression patterns [24, 25]. While it has been demonstrated that the majority of lncRNAs are localized in the nucleus, some have also been shown to reside in the cytoplasm. In the nucleus, lncRNAs are involved in RNA splicing, transcriptional regulation, epigenetic modifications, and chromatin organization, whereas they regulate subcellular transport of ribonucleoprotein complexes, RNA stability, translational efficiency, and gene expression at post-transcriptional levels in the cytoplasm [22, 25]. To summarize the mechanisms of these actions of lncRNAs, four classes have been proposed: decoy, scaffold, signal, and guide. Decoy lncRNAs act as competitive molecules and prevent their binding to transcriptional regulators, specifically miRNAs. Scaffold lncRNAs mediate the physical interactions between noncoding RNAs and proteins to assist regulatory complex formation. Signal lncRNAs act as molecular indicators to reflect specific biological conditions, while guide lncRNAs recruit the transcription factors to specific genomic sites [26].

## 4 LncRNAs MODULATE AUTOPHAGY IN NEUROLOGICAL DISEASES

Neurodegenerative disorders are now a significant global public health issue with a significant economic burden. Within 20 years, these disorders will surpass cancer as the second most common cause of mortality, according to the WHO prediction. Therefore, finding pathogenic and diagnostic molecular markers for neurodegenerative disorders is urgently needed. There is evidence that defects in the autophagy process are associated with neurodegenerative disorders. For instance, studies have reported that Beclin-l levels remarkably reduced in Alzheimer's disease (AD) [27, 28]. Indeed, Beclin-1 overexpression and, subsequently, autophagy activation improves the clearance of aggregated toxic proteins and prevents neuronal cell death [29]. In Parkinson’s disease (PD), autophagy plays neuroprotective roles by preventing α-synuclein toxicity, a protein that aggregates in PD [30]. Among the various elements affecting autophagy during neurological disorders, lncRNAs are one of the critical regulatory molecules. According to studies, the brain expresses nearly 40% of all lncRNAs [31-33]. Brain lncRNAs are more strongly temporally and spatially specific than protein-coding genes and are highly conserved [32]. In a study, Wang *et al.* indicated that overexpression of lncRNA17A suppressed autophagy and neurogenesis in AD cell lines, whereas its downregulation reduced apoptosis as well as invasion and migration of cells [34]. Another study concluded that moxibustion, a traditional Chinese medicine therapy that involves burning dried mugwort on particular points on the body, suppresses lncRNA SIX homeobox 3, opposite strand 1 (SIX3OS1) expression in a mouse model of AD, in which SIX3OS1 silencing improved clearance of Aβ1-42 by promoting autophagy through the inactivation of PI3K/AKT/ mTOR pathway [35]. Table **1** summarizes the modulatory effects of lncRNA dysregulation during neurological disorders and their effects on the autophagy process [36-44].

## 5 LncRNAs MODULATE AUTOPHAGY IN ISCHEMIC STROKE

There is growing evidence that the expression of various lncRNAs is dysregulated during ischemic stroke, modulating different cellular processes (Table **2**). For instance, stroke disrupts the body's oxidative balance and triggers the production of additional reactive oxygen species (ROS), causing oxidative stress damage [45]. LncRNAs can modulate ROS production during ischemic stroke, and their targeting is a reliable strategy to attenuate the pathophysiological condition. Wang *et al.* indicated that the elevated levels of lncRNA AK046177, miR-134, and ROS during ischemic stroke were attenuated by safflor yellow B, which finally led to the upregulation of nuclear factor erythroid 2-related factor 2 (Nrf2) and reduction in ischemic stroke injury [46]. It is worth noting that Nrf2 is a transcription factor that upregulates vitagenes, including heat shock proteins, NAD(P)H quinone oxidoreductase 1 (NQO1), glutathione reductase, and glutathione S-trans-ferases (GSTs) [47, 48]. Vitagenes are protective genes that preserve cellular homeostasis under stressful conditions [47]. This system provides stress-resistant properties to neuronal cells that prevent neurodegeneration [49]. For example, there is evidence that curcumin exerts its neuroprotective effects on neuro-inflammation by promoting the vitagene system and Nrf2, leading to the suppression of NF-κB and creating a balance between pro-inflammatory and anti-inflammatory molecules [50]. In this section, we will focus on how autophagy is regulated during ischemic stroke by lncRNAs. Fig. (**2**) summarizes several dysregulated lncRNAs during ischemic stroke and their regulatory effect on autophagy (Table **2**) [51-62].

### 5.1 LncRNA MALAT1

Metastasis-associated lung adenocarcinoma transcript 1 (MALAT1) gene is found on chromosome 19qA in mice, with a 6.7-kb length transcript, and chromosome 11q13 in humans, with a 7-kb length transcript [63]. There is evidence that lncRNA MALAT1 is involved in various physiological conditions, such as neural development and functions, skeletal myogenesis, and vascular growth, as well as pathological conditions, including cancer, diabetic retinopathy, cardiovascular diseases, Parkinson's disease, Alzheimer's disease, and ischemic stroke. In A-25-35-induced rat models and cells, miR-30b was expressed at a high level, and lncRNA MALAT1 and cannabinoid receptor 1 (CNR1) were expressed at insufficient levels, whereas the upregulation of MALAT1 and CNR1 increased the viability of neuronal cells and lessened neurological damage in the hippocampus of rats. MALAT1 might act as a sponge for mR-30b to increase the expression of CNR1 [64]. To mimic ischemia-reperfusion injury *in vitro*, Li *et al.* used oxygen-glucose deprivation/reoxygenation (OGD/R) and investigated the role of MALAT1 in brain microvascular endothelial cell (BMEC) injury. They concluded that MALAT1 upregulation activated the autophagy of BMECs and promoted cellular survival against OGD/R-induced injury. Mechanistically, MALAT1 downregulated the expression of miR-26b by directly interacting with and sponging miR-26b. Additionally, MALAT1 reversed miR-26b's inhibitory effect on the survival and autophagy of BMECs, which was involved in the upregulation of ULK2, as a target of miR-26b. Overall, MALAT1 plays a protective role in I/R by promoting the survival and autophagy of BMECs through the MALAT1/miR-26b/ULK2 axis [65]. Consistent with these findings, Wang *et al.* demonstrated that MALAT1 protects BMEC against OGD-induced injury by inducing autophagy. They showed that OGD condition leads to the downregulation of p62 and upregulation of MALAT1 and LC3BII, while MALAT1 inhibition suppressed autophagy and encouraged cell death. In a mechanistic view, MALAT1 increases the expression of silent information regulator 1 (SIRT1) by binding to and negatively regulating miR-200c-3p [66]. SIRT1 is a histone deacetylase, and its protective role during neurological diseases, such as ischemia, and autophagy-inducer activities, has been demonstrated [67, 68]. In another study, Guo *et al.* reported the upregulated levels of lncRNA MALAT1 and Beclin-1 and increased conversion of LC3-I to LC3-II as well as downregulation of miR-30a in cerebral cortex neurons after OGD and mouse brain cortex after middle cerebral artery occlusion-reperfusion. Furthermore, MALAT1 downregulation inhibited autophagy activation and suppressed ischemic injury due to the binding of MALAT1 to miR-30a [69]. Therefore, MALAT1 regulates autophagy during ischemic stroke through the MALAT1/miR-26b/ULK2, MALAT1/moR-200c-3p/SIRT1, and MALAT1/miR-30a/Beckin-1 axes.

### 5.2 LncRNA MIAT

The lncRNA myocardial infarction-associated transcript (MIAT) is located on chromosome 22q12, and its higher levels of expression have been reported in the nervous system [70]. There is evidence that lncRNA is dysregulated in various pathological conditions, such as cancers, bone diseases, diabetic nephropathy, cardiomyopathy, Parkinson’s disease, and ischemic stroke. In Parkinson’s disease rats, MIAT downregulation was observed in the striatum, substantia nigra, hippocampus, and cortex compared with normal rats, and its upregulation was associated with increased cellular viability and decreased apoptosis, proposing the neuroprotective role of lncRNA MIAT in Parkinson’s disease [71]. Regarding ischemic stroke, Zhang *et al.* indicated that lncRNA MIAT upregulated in ischemic stroke patients alongside the upregulation of interleukin (IL) 1B and downregulation of miR-874-3p. A middle cerebral artery occlusion model in rats revealed that MIAT knockdown suppresses neuronal apoptosis and improves neurological functions, suggesting that MIAT impairs neurological functions in ischemic stroke by regulating miR-874-3p and IL1B [55]. Moreover, lncRNA MIAT is considered an unfavorable biomarker in ischemic stroke and response to therapeutics, in which lower levels of MIAT were reported in patients with promising outcomes. Importantly, patients with higher levels of MIAT exhibited a higher risk of death [72]. To clarify the role of MIAT in ischemic stroke and explore its mechanism of action, Guo *et al.* investigated the expression of lncRNA MIAT as well as apoptotic- and autophagic-related proteins in OGD/R-induced PC12 cell injury and ischemic stroke rat models. They found that the lncRNA MIAT and regulated in development and DNA damage response 1 (REDD1) expressions were upregulated in both the cellular model and rat model of ischemic stroke. The results of flow cytometry revealed that the rate of apoptosis was decreased following interference with si-MIAT, and according to the results of the western blotting, p-mTOR, p62, and Bcl-2 expression were upregulated while LC3II/LC3I, Bax, and cleaved-caspase3 were downregulated. RNA-binding protein immunoprecipitation (RIP) assay revealed that MIAT promotes REDD1 expression by binding to it, and interference with si-MIAT accelerates REDD1 degradation in a ubiquitin-dependent manner. Therefore, MIAT stimulates apoptosis and autophagy of neural cells and exacerbates ischemic stroke by increasing REDD1 expression [73]. It is worth noting that REDD1 is an endogenous inhibitor of mTOR, which regulates cellular response to various stressful conditions [74].

### 5.3 LncRNA SNHG12

Small nucleolar host gene 12 (SNHG12), with approximately 1,867 bases long, is located on chromosome 1p35.3, and its expression is dysregulated in different diseases, such as cancer, diabetes, pulmonary arterial hypertension, and neurological disorders [75]. There is accumulating evidence that lncRNA SNHG12 plays neuroprotective roles in cerebral ischemic injury. The knockdown of lncRNA SNHG12 reduces cell viability and aggravates OGD/R-induced apoptosis, alongside an increase in the levels of pro-inflammatory cytokines [76]. In contrast to SNHG12 knockdown, SNHG12 overexpression prevents inflammatory response and BMEC death while promoting angiogenesis following OGD/R [77]. In another study, Zhao *et al.* also demonstrated the pro-angiogenesis ability of lncRNA SNHG12 following ischemic stroke. They concluded that the expression of SNHG12 was upregulated and the expression of miR-150 was downregulated in OGD-exposed brain endothelial cells, whereas knockdown of SNHG12 inhibited capillary tubing, migration, viability, and vascular endothelial growth factor (VEGF) expression. Moreover, elevated levels of SNHG12 promoted the recovery of neuronal function, decreased infarct volume and miR-150 expression, and increased vessel density and VEGF levels in the mice model of ischemic stroke, proposing that SNHG12 acts as a neuroprotective lncRNA in ischemic stroke through the stimulation of angiogenesis and regulation of SNHG12/miR-150/VEGF axis [60]. In addition to pro-angiogenesis activity, lncRNA SNHG12 also exerts its neuroprotective function through the induction of autophagy. For example, Yao *et al.* reported the upregulation of SNHG12 in cellular and mouse models of ischemic stroke. Increased LC3 II/I ratio and Beclin-1 and a decrease in p62 are indications that up-regulated SNHG12 induced autophagy activation and reduced cellular injury during ischemic stroke. Therefore, lncRNA SNHG12 reduces cerebral ischemic stroke injury by activating autophagy [78]. Also, SNHG12's function as a regulator of mesenchymal stem cell (MSC) activity in ischemic stroke injury is established. It was discovered that SNHG12 downregulation increased MSCs' ability to inhibit autophagy *via* the PI3K/AKT/mTOR axis. Autophagy, apoptosis, and infarcted areas were decreased in ischemic stroke injury treated with SNHG12-modified MSCs [79].

### 5.4 LncRNA H19

The *H19* gene is located on human chromosome 11p15.5 and mouse chromosome 7. The transcription of the H19 gene by RNA polymerase II leads to the production of a 2.3 kb lncRNA, which is then transported from the nucleus to the cytoplasm [80]. In addition to its pivotal roles in physiological conditions such as angiogenesis during embryonic development, lncRNA H19 is involved in several pathological conditions, including cancer, cerebral hemorrhage, Parkinson's disease, Alzheimer's disease, spinal cord injury, neuropathic pain, temporal lobe epilepsy, and ischemic stroke. Compared to healthy controls, the level of circulating H19 was considerably higher in ischemic stroke patients, demonstrating high diagnostic specificity and sensitivity. In addition, plasma H19 levels were positively correlated with tumor necrosis factor-α (TNF-α) levels and National Institutes of Health Stroke Scale scores. The administration of si-H19 decreased IL-1β and TNF-α levels, increased IL-10 concentrations in plasma, and reduced infarct size and brain edema [81]. The pro-inflammatory properties of lncRNA H19 in ischemic stroke were demonstrated by Li *et al.*, who showed that H19 stimulated inflammation by leukocytes during an ischemic stroke by acting as a sponge for miR-29b to upregulate C1q and tumor necrosis factor 6 (C1QTNF6). The elevated levels of C1QTNF6 induced the production of TNF-α and IL-1β in leukocytes, leading to the disruption of the blood-brain barrier [82]. Additionally, the rs217727 variant in H19 was linked to ischemic stroke susceptibility and a higher risk of ischemic stroke [83]. To explore the relationship between lncRNA H19 and its mutations and autophagy activation in ischemic stroke, Wang *et al.* induced cerebral ischemia and reperfusion injury in SH-SY5Y cells by OGD/R and in rats by middle cerebral artery occlusion followed by reperfusion. They found that lncRNA H19 expression was increased in both *in vitro* and *in vivo* models of ischemic stroke. Moreover, diminishing lncRNA H19 expression and inhibiting autophagy prevented OGD/R-induced cell death, proposing that lncRNA H19 overexpression and autophagy activation could stimulate cellular apoptosis during ischemic stroke. Due to the activation of autophagy alongside the upregulation of lncRNA H19, they concluded that lncRNA H19 damages cell viability by activating the autophagy process during ischemic stroke. In a mechanistic view, lncRNA H19 stimulated autophagy activation by inhibiting dual-specificity protein phosphatase 5 (DUSP5) and modulating the DUSP5-ERK1/2 axis [84]. Indeed, DUSP5 is a mitogen-activated protein kinase phosphatase (MKP), and its upregulated levels lead to the inhibition of autophagy through the inhibition of ERK1/2 [85]. According to the mentioned studies, lncRNA H19 can be considered a new potential therapeutic candidate for ischemic stroke.

### 5.5 LncRNA AC136007. 2

Li *et al.* conducted an investigation to discover the expression profile of lncRNAs in acute ischemic stroke and their mechanism of action as well as their therapeutic potential in the treatment of the disease by real-time quantitative polymerase chain reaction (qRT-PCR) and high-throughput RNA sequencing (RNA-Seq) and then pathway and network analyses using Kyoto Encyclopedia of Genes and Genomes (KEGG) and Gene Ontology (GO) databases. According to the analysis of RNA-Seq, 4,263 mRNAs and 791 lncRNAs were downregulated, whereas 957 mRNAs and 428 lncRNAs were upregulated in acute ischemic stroke patients compared with normal ones. The oxidative, inflammatory, apoptosis and calcium signaling pathways were potentially involved in the pathology of acute ischemic stroke, according to GO enrichment and KEGG pathway analyses. Additionally, lncRNA AC136007.2 and lncRNA C14orf64 were remarkably downregulated in the blood sample of patients with acute ischemic stroke and considered valuable markers for the diagnosis of acute ischemic stroke [86]. In another study, Liu *et al.* tried to explore the role and function of lncRNA AC136007.2 in ischemic stroke by modeling the disease both *in vitro* and *in vivo*. In the cellular model of ischemic stroke, the expression levels of lncRNA AC136007.2 were decreased along with decreased cellular viability, induced apoptosis, and increased secretion of IL-1β, IL-6, and TNF-α. In contrast, these phenomena were then considerably reversed by lentiviral-mediated AC136007.2 upregulation. Further analyses revealed that lncRNA AC136007.2 suppresses autophagy by inactivating the AMPK/mTOR pathway, characterized by increased phosphorylation of AMPK, decreased phosphorylation of mTOR and downregulation of Beclin-1 and LC3-I/II. In a rat model of ischemic stroke, intraventricular lncRNA AC136007.2 administration led to reduced cerebral infarction and brain edema [87]. Therefore, lncRNA AC136007.2 improves ischemic stroke through the suppression of autophagy in an AMPK/mTOR-dependent manner.

### 5.6 LncRNA C2dat2

It has been shown that Ca^2+^/calmodulin-dependent protein kinase II (CaMKII) plays a role in ischemic stroke [88]. Ye *et al.* identified that the expression of CAMK2D/ CaMKIIδ and CAMK2G/CaMKIIγ were elevated in ischemic stroke, and both CaMKIIδ and CAMKIIγ were involved in neuronal survival. The expression of CaMKIIδ is increased by ischemic stroke primary neurons dependent on two CAMK2D-associated lncRNAs (C2dat1 and C2dat2). C2dat1/2 specifically targets CAMK2D, as evidenced by the fact that C2dat1/2 knockdown prevented OGD/R from inducing CaMKIIδ expression and reduced neuronal survival without altering CaMKIIγ levels. By upregulating IKKα/β and further activating NF-κB signaling, ischemic stroke-induced CaMKIIδ and CaMKIIγ prevented neurons from suffering ischemic injury [89]. In another study, Guo and Kan investigated the mechanism of action of lncRNA C2dat2 in regulating cerebral ischemia-reperfusion injury. A mice model of cerebral ischemia-reperfusion injury revealed that the expression levels of lncRNA C2dat2 and DNA damage-inducible transcript 4 (DDIT4) were increased, whereas miR-30d-5p expression exhibited lower levels compared with healthy tissues, suggesting that C2dat2 acts as a sponge for miR-30d-5p to upregulate DDIT4. The knockdown of lncRNA C2dat2 not only suppressed OGD-induced neuronal apoptosis but also inhibited autophagy, characterized by a decrease in p-P70S6K/P70S6K ratio, p-mTOR/ mTOR ratio, and p62 as well as an increase in LC3B II/I ratio and Beclin-1 levels. Therefore, lncRNA C2dat2 is upregulated in cerebral ischemia-reperfusion injury, and it promotes apoptosis and autophagy through the miR-30d-5p/DDIT4/mTOR axis [90]. Recently, Zhang *et al.* also found that autophagy is activated following cerebral ischemia-reperfusion injury by regulating the AMPK/DDIT4/mTOR pathway [91].

### 5.7 LncRNA MEG3

Maternally expressed gene 3 (MEG3) is located on human chromosome 14q32 and mouse chromosome 12 [92]. There is growing evidence that lncRNA MEG3 expression is dysregulated during various diseases, including cancers, Huntington's disease, Parkinson's disease, cardiovascular diseases, bone diseases, and ischemic stroke. For instance, MEG3 is upregulated after OGD/R, and it suppresses neural stem cell proliferation and promotes the apoptosis of vascular cells [93, 94]. Xiang *et al.* reported that lncRNA MEG3 and semaphorin-3A (Sema3A) were upregulated, whereas miR-424-5p was underexpressed in ischemic stroke samples, according to the findings of bioinformatic analysis. Moreover, MEG3 inhibition significantly downregulated Sema3A, upregulated miR-424-5p, and activated the MAPK pathway, as well as decreased cellular apoptosis and increased cell viability in the OGD/R model [95]. In another study, Luo *et al.* demonstrated that lncRNA MEG3 induces autophagy in ischemic stroke through the MEG3/miR-378/growth factor receptor-bound protein 2 (GRB2) axis. Higher levels of MEG3 in ischemic stroke lead to the upregulation of GRB2 by binding to and inhibiting miR-378, resulting in the destruction of the Akt/mTOR pathway. They also exhibited that miR-378 prevented neuronal death and neurological functional impairment by silencing GRB2 in mice, as well as neuronal loss and autophagy [96]. Similarly, the upregulation of MEG3 and its positive effects on autophagy activation in HT22 cells treated with OGD/R was reported, while MEG3 knockdown remarkably inhibited autophagy. Mechanistically, lncRNA MEG3 acts as a sponge for miR-181c-5p to increase the levels of ATG7. Furthermore, an *in vivo* model of ischemic stroke revealed that the knockdown of MEG3 not only inhibited the activation of autophagy but also reduced infarct size and ameliorated behavioral deficits [97]. Taken together, lncRNA MEG3 is overexpressed in ischemic stroke and promotes autophagy, thus, targeting MEG3 expression can attenuate ischemic stroke damage by inhibiting autophagy.

### 5.8 LncRNA KCNQ1OT1

KCNQ1 Opposite Strand/Antisense Transcript 1 (KCNQ1OT1), called LIT1 or KCNQ1 overlapping transcript 1, is located on human chromosome 11p15.5 with 91 kb length [98]. Recent studies have shown that various human diseases exhibit aberrant KCNQ1OT1 expression, such as various cancers, osteoarthritis and other bone-related diseases, atherosclerosis, retina disease, myocardial infarction, diabetic cardiomyopathy, and neuronal diseases. For instance, KCNQ1OT1 inhibition can reduce neuronal apoptosis and neuroinflammation by controlling the miR-30e-3p/NLRP3 axis, indicating that KCNQ1OT1 and miR-30e-3p may be promising therapeutic targets for the treatment of neurological disorders [99]. Regarding ischemic stroke, there is evidence that lncRNA KCNQ1OT1 promotes neuronal injury. Ren *et al.* showed that KCNQ1OT1 levels were increased after inducing OGD/R models in neuronal cells, whereas KCNQ1OT1 knockdown reduced neuronal damage. Mechanistically, KCNQ1OT1 increased the expression of matrix metalloproteinase 8 (MMP8) by binding to and inhibiting miR-9 [100]. Lower triglyceride levels are associated with more severe strokes, and MMP8 is related to a reduced level of triglyceride in ischemic stroke patients [101]. KCNQ1OT1 also mediates ischemic stroke-induced cellular damage by acting as a sponge for miR-153-3p, which leads to the upregulation of Forkhead box O3a (Foxo3) [102]. In another study, Yu *et al.* investigated the expression of lncRNA KCNQ1OT1 in ischemic stroke and tried to explore its mechanism of action. They indicated that KCNQ1OT1 expression was elevated in ischemic stroke, while its knockdown lessened brain injury and suppressed autophagy in a mice model of ischemic stroke. In a mechanistic view, KCNQ1OT1 positively regulated the expression of Foxo3 by sponging miR‐200a. Additionally, Foxo3 induced autophagy in ischemic stroke damage by directly promoting the expression of ATG7 as a transcription factor [103].

### 5.9 LncRNA SNHG3

Small nucleolar RNA host gene 3 (SNHG3) is located on human chromosome 1p35.3 with a length of around 2.3 kb [104], which is involved in cellular proliferation, apoptosis and the development of various diseases, including cardiovascular diseases, different cancers, Alzheimer's disease, and ischemic stroke. For instance, Yang *et al.* reported the downregulated levels of lncRNA SNHG3 in hypoxic-ischemic brain damage to neonates, while its overexpression could protect brain damage in both *in vitro* and *in vivo* models of hypoxic-ischemic. In the hippocampal cells, SNHG3 binds to miR-196 to increase the expression of target genes of miR-196, such as caspase activity and apoptosis inhibitor 1 (CAAP1) and X-chromosome-linked inhibitor of apoptosis protein (XIAP), which are involved in cellular apoptosis [105]. In another study, Huang *et al.* showed that the expressions of SNHG3, Ionized calcium-binding adaptor molecule 1 (Iba1) as a marker of microglial activation, and IL-6 and TNF-α as pro-inflammatory markers were elevated in *in vitro* and *in vivo* models of cerebral ischemia-reperfusion injury. Moreover, SNHG3 increased the expression of histone deacetylase 3 (HDAC3) by binding to and stabilizing HDAC3, which inhibits its ubiquitination and degradation [106]. There is evidence that HDAC3 stimulates the formation of pro-inflammatory microenvironment by regulating the cyclic GMP-AMP synthase (cGAS)-stimulator of the interferon genes (cGAS-STING) pathway, whereas the inhibition of HDAC3 attenuates cerebral ischemia/reperfusion-induced brain damage and neuroinflammation [107]. Sun *et al.* indicated the reduced expression of miR-302a-3p in the brain of a mice model of cerebral ischemic stroke, whereas the upregulation of miR-302a-3p not only decreased the levels of pro-inflammatory markers but also increased the number of neurons and improved nerve repair. Mechanistically, E2F1 was the target of miR-302a-3p, and E2F1 triggered the transcription of SNHG3, thus, SNHG3 or E2F1 reversed the neuronal repair properties of miR-302-3p [108]. Regarding the effects of lncRNA SNHG3 on autophagy during ischemic stroke, Cao *et al.* examined the expression of SNHG3, miR-485, and ATG7 in OGD-treated N2a cells and a mice model of cerebral ischemic stroke. They found that in contrast to miR-485, the expression of lncRNA SNHG3 was upregulated in ischemic stroke models, leading to increased levels of Beclin-1 and LC3-II/LC3-I ratio. The raised expression of SNHG3 induced apoptosis and autophagy. In a mechanistic view, lncRNA SNHG3 acted as a sponge for miR-485 to increase the level of ATG7. Therefore, lncRNA SNHG3 acts as a ceRNA for miR-485 to upregulate ATG7, which in turn stimulates autophagy-induced neuronal cell apoptosis [109].

### 5.10 LncRNA RMRP

RNA component of mitochondrial RNA processing endoribonuclease (RMRP) is located on human chromosome 9p13.3 [110], and its expression is dysregulated in several pathological conditions, including cancer, human cartilage-hair hypoplasia, coronary atherosclerosis, rheumatoid arthritis, major depressive disorder, and ischemic cerebral injury. For example, Li and Sui demonstrated that valproate (VPA), by altering the lncRNA RMRP/PI3K/AKT pathway, could attenuate cerebral ischemic stroke-induced damages in microglia and mice. VPA not only inhibited apoptosis and reduced infarction size but also suppressed the expression of lncRNA RMRP and prevented the activation of PI3K/AKT signaling [111]. Due to the stimulatory effects of Schwann cells (SCs) on the regeneration of nerves following facial nerve injury, Zhou *et al.* tried to discover the function of RMRP in SCs. In contrast to RMRP overexpression, silencing of RMRP promoted SC cell proliferation and migration. On a mechanistic level, by sponging miR-766-5p, lncRNA RMRP positively controlled cullin-associated and neddylation-dissociated 1 (CAND1) expression. These findings proposed that RMRP was considerably involved in SC migration and proliferation, suggesting that it might be a promising target for facial nerve injury treatment [112]. To explore the modulatory effects of lncRNA RMRP on apoptosis and autophagy in OGD/R-induced neuronal damage, Zhou *et al.* evaluated its expression levels and mechanism of action in OGD/R-induced injury in SH-SY5Y cells. They indicated that lncRNA RMRP expression was dramatically increased in the OGD/R treated cells, whereas the knockdown of lncRNA RMRP inhibited apoptosis and G1 cell cycle arrest of the cells. Since OGD/R induces the activation of autophagy in SH-SY5Y cells, RMRP knockdown reversed the autophagy process. The inhibition of LC3II, p-mTOR, p-PI3K, and p-Akt as well as the induction of Bcl-2 and P62 was linked to the effects of RMRP suppression on the phenotypes of SH-SY5Y. Thus, the PI3K/Akt/mTOR-mediated apoptosis and the activation of autophagy may both play a role in how RMRP functions during I/R injury [113].

## CONCLUSION

In terms of public health concerns, only cancer and cardiovascular diseases rank higher than stroke, and it is the second and third cause of death and disability, respectively. Although most of the patients can recover from ischemic stroke thanks to efficient treatments, the majority of them experience sequelae like cognitive decline, language barrier, swallowing disorder, depression, and hemiplegia. Additionally, the recurrence risk is 30% at five years for ischemic stroke. Therefore, it is crucial to excavate and explore new therapeutic targets for ischemic stroke. Autophagy plays a crucial role in ischemic stroke and can be controlled with different important signaling pathways, such as the lncRNA/miRNA/autophagy axis. Our primary objective was to build a network between the lncRNA/miRNA/autophagy axis and ischemic stroke and their crosstalk for providing insight into identifying reliable biomarkers and effective therapeutic candidates. To use the crosstalk between the lncRNA/miRNA/autophagy axis and ischemic stroke in introducing diagnostic biomarkers and therapeutic targets, some issues must be addressed, including the time of using these biomarkers due to the complexity of ischemic stroke, identifying that the dysregulation of lncRNAs occurs in the initial stage or progression of ischemic stroke, determining the delivery platform of lncRNA silencing approaches, clarifying the accurate role of autophagy in ischemic stroke, elucidating the specific mechanism of action of autophagy-inducers or autophagy-inhibitors and their cytotoxic effects, and balancing the properties of autophagy process in inducing cell survival or cell death. Therefore, the crosstalk between the lncRNA/miRNA/autophagy axis and ischemic stroke is still in its infancy and requires more investigation.

## Figures and Tables

**Fig. (1):**
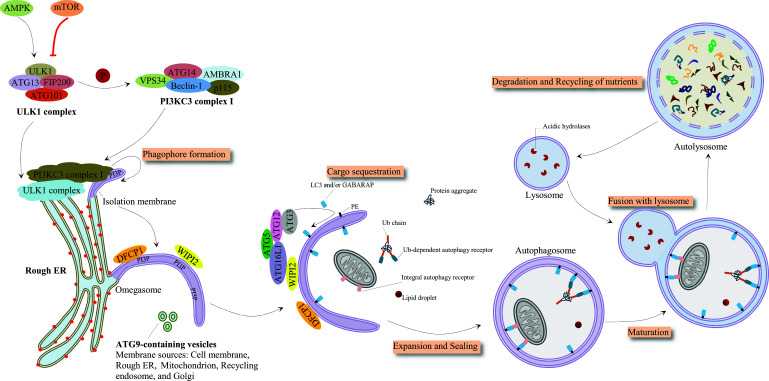
Molecular mechanism of autophagy. Under sufficient nutrients and growth factors conditions, mTOR inhibits the formation of ULK1 complex by ATG13 phosphorylation. Under low cytosolic ATP levels and hypoxia, AMPK promotes the activation of ULK1 complexes and downregulates mTOR activity, leading to phagophore formation by recruiting and activating PI3KC3 complex I, which increases PI3P and its interaction with DFCP1 and WIPI2. Two ubiquitin-like conjugation systems are involved in the elongation and maturation of phagophore and, subsequently, in the formation of autophagosome: ATG5-ATG12-ATG16L1 complex and LC3 processing system. The cargo-carrying autophagosome fuses with the lysosome, resulting in the degradation of cargo and recycling of nutrients.

**Fig. (2):**
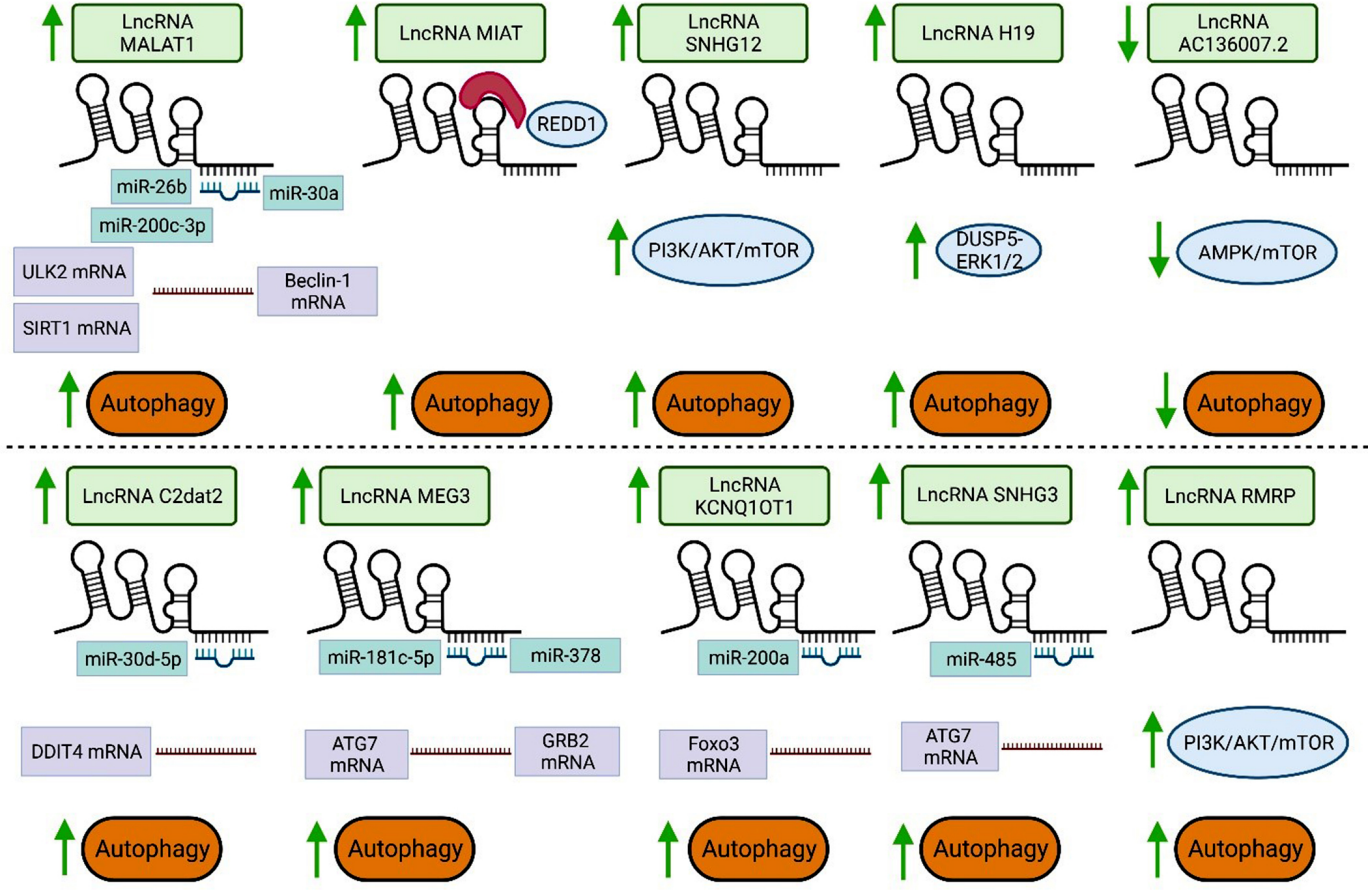
The modulatory effects of lncRNAs on autophagy during ischemic stroke. LncRNAs could regulate the autophagy process in ischemic stroke by modulating the lncRNA/miRNA/mRNA networks and signaling pathways, as well as the expression of proteins.

**Table 1:** Dysregulation of lncRNAs during neurological disorders and their effects on the autophagy process.

**Disorder**	**LncRNA**	**Autophagy Axis**	**References**
Alzheimer's Disease	↑BACE1-AS	miR-214-3p/ATG5	[36]
	↑RMRP	miR-3142/TRIB3	[37]
Parkinson's disease	↑SNHG1	miR-221/222/p27/mTOR	[38]
	↑BDNF-AS	miR-125b-5p	[39]
	↑NEAT1	miR-374c-5p	[40]
Brain tumors	↑TPT1-AS1	miR-770-5p/STMN1	[41]
	↑MALAT1	miR-101/STMN1/RAB5A/ATG4D	[42]
	↓CASC2	miR-193a-5p/mTOR	[43]
Spinal cord injury	↑TSIX	miR-1283/TP53INP2	[44]

**Note:** ATG5, autophagy related 5; TRIB3, tribbles pseudokinase 3; mTOR, mammalian target of rapamycin;STMN1, stathmin1; TP53INP2, tumor protein p53 inducible nuclear protein 2.

**Table 2:** Modulatory effects of lncRNAs dysregulation on cellular processes during ischemic stroke.

**LncRNA**	**Up/Downregulated**	**miRNA/mRNA Axis**	**Cellular Process**	**References**
GAS5	Upregulated	miR-137/Notch1	↓Cellular viability	[51]
TUG1	Upregulated	miR-9/Bcl2l11	↑Neuron apoptosis	[52]
PVT1	Upregulated	miR-24-3p/STAT3	↑Neuron apoptosis ↑Oxidative stress	[53]
RMST	Upregulated	miR-221-3p/PIK3R1	↑Oxidative stress ↑Inflammation	[54]
MIAT	Upregulated	miR-874-3p/IL1B	↑Neuron apoptosis ↑Inflammation	[55]
SNHG15	Upregulated	miR-18a/CXCL13	↑Neuron apoptosis	[56]
FGD5-AS1	Downregulated	miR-223/IGFIR	↑Neuron apoptosis	[57]
FTX	Downregulated	miR-342-3p/SPI1	↓Cellular viability ↓Angiogenesis	[58]
MEG8	Upregulated	miR-130a-5p/VEGFA	↑Angiogenesis	[59]
SNHG12	Upregulated	miR-150/VEGFA	↑Angiogenesis	[60]
PEG11as	Upregulated	miR-342-5p/PFN1	↑Neuron apoptosis	[61]
ANRIL	Upregulated	miR-671-5p/NF-κB	↑Inflammation	[62]

## LIST OF ABBREVIATIONS

AD: Alzheimer's disease
BMEC: Brain Microvascular Endothelial Cell
IL: Interleukin
lncRNAs: Long Non-coding RNAs
ncRNAs: Noncoding RNAs
OGD/R: Oxygen-glucose Deprivation/Reoxygenation
PD: Parkinson’s Disease
TNF-α: Tumor Necrosis Factor-α
ULK1: Uncoordinated-51-like Protein Kinase
